# Increased Abundance of Tumour-Associated Neutrophils in HPV-Negative Compared to HPV-Positive Oropharyngeal Squamous Cell Carcinoma Is Mediated by IL-1R Signalling

**DOI:** 10.3389/froh.2021.604565

**Published:** 2021-02-11

**Authors:** Sarmad Al-Sahaf, Naeima B. Hendawi, Bethany Ollington, Robert Bolt, Penelope D. Ottewell, Keith D. Hunter, Craig Murdoch

**Affiliations:** ^1^School of Clinical Dentistry, University of Sheffield, Sheffield, United Kingdom; ^2^Department of Oncology and Metabolism, University of Sheffield, Sheffield, United Kingdom

**Keywords:** oropharyngeal cancer, human papillomavirus, leukocytes, fibroblasts, chemokine, IL-1, neutrophils

## Abstract

The incidence of human papillomavirus (HPV)-associated cancer is increasing and HPV is now implicated in the aetiology of more than 60% of all oropharyngeal squamous cell carcinomas (OPSCC). In OPSCC, innate immune cells such as neutrophils and macrophages generally correlate with poor prognosis, whilst adaptive immune cells, such as lymphocytes, tend to correlate with improved prognosis. This may, in part, be due to differences in the immune response within the tumour microenvironment leading to the recruitment of specific tumour-associated leukocyte sub-populations. In this study, we aimed to examine if differences exist in the levels of infiltrated leukocyte sub-populations, with particular emphasis on tumour-associated neutrophils (TAN), and to determine the mechanism of chemokine-induced leukocyte recruitment in HPV-positive compared to HPV-negative OPSCC. Immunohistochemical analysis showed that HPV-negative OPSCC contained significantly more neutrophils than HPV-positive tumours, whilst levels of CD68+ macrophages and CD3+ lymphocytes were similar. Using a 3D tissue culture model to represent tumour-stromal interactions, we demonstrated that HPV-negative tumour-stromal co-cultures expressed significantly higher levels of CXCL8, leading to increased neutrophil recruitment compared to their HPV-positive counterparts. HPV-negative OPSCC cells have previously been shown to express higher levels of IL-1 than their HPV-positive counterparts, indicating that this cytokine may be responsible for driving increased chemokine production in the HPV-negative 3D model. Inhibition of IL-1R in the tumour-stromal models using the receptor-specific antagonist, anakinra, dramatically reduced chemokine secretion and significantly impaired neutrophil and monocyte recruitment, suggesting that this tumour-stromal response is mediated by the IL-1/IL-1R axis. Here, we identify a mechanism by which HPV-negative OPSCC may recruit more TAN than HPV-positive OPSCC. Since TAN are associated with poor prognosis in OPSCC, our study identifies potential therapeutic targets aimed at redressing the chemokine imbalance to reduce innate immune cell infiltration with the aim of improving patient outcome.

## Introduction

The worldwide increasing incidence of human papillomavirus (HPV)-driven oropharyngeal squamous cell cancer (OPSCC) has raised the profile of these tumours and intensified interest in this area of cancer research [1]. Of particular interest is the finding that HPV-positive OPSCC generally display improved prognosis and response to therapy than their HPV-negative counterparts, whose aetiology is generally linked to DNA damage by classical risk factors such as tobacco use and alcohol consumption rather than viral infection [2]. In addition to aetiology, the tumour microenvironment also has a profound influence on tumour progression. The tumour microenvironment consists of dynamic molecular interactions between cancer cells, fibroblasts, the extracellular matrix and immune cell populations, where pro-tumour factors outweigh those intended to inhibit disease.

In many malignant tumours, a pro-inflammatory tumour microenvironment drives the recruitment of tumour-infiltrating leukocytes that in turn have a major effect on tumour progression [3]. The presence of increased tumour-associated neutrophils (TAN) and tumour-associated macrophages (TAM) have been correlated with poor clinical outcome in many tumours, whilst on many occasions tumour-infiltrating lymphocytes (TIL) have been associated with improved prognosis [4]. There is good evidence to suggest that HPV-positive OPSCC contain abundant CD8+ T-cells that can recognise tumour cell-expressed HPV antigens, enabling activation of adaptive immune responses to eliminate cancer cells and impart improved outcome [5–7]. Although increased levels of TAM and TAN correlate with poor prognosis in oral squamous cell carcinoma (OSCC) [8, 9], the levels of TAM in HPV-positive/negative OPSCC are less well-characterised and to date there are no published studies correlating abundance of TAN in OPSCC with HPV status.

It has been known for many years that the increased gene expression and subsequent secretion of chemokines by cells at infected or inflammatory sites is mediated by pro-inflammatory cytokines, such as IL-1, that are present within the local environment [10]. There are 47 different human chemokines each with specific or overlapping affinities for different leukocyte populations [11]. Leukocytes are recruited to inflammatory sites and tumours from the circulation via chemotactic gradients of chemokines. Although OPSCC cells are known to over-express a number of chemokines, many now believe that the pro-inflammatory paracrine signalling between tumour cells and the surrounding tumour-associated fibroblasts is the driving force behind elevated chemokine expression in the tumour microenvironment [12]. The specific type and proportions of leukocytes recruited to tumours are directly related to the profile of chemokines released. We have recently demonstrated that HPV-negative OPSCC cells stimulate tonsillar fibroblasts to secrete a distinct chemokine-rich profile compared to HPV-positive OPSCC cells. Moreover, this effect is mediated by the actions of tumour cell-secreted IL-1 on the IL-1R expressed by tonsillar fibroblasts [13]. It is therefore plausible that the chemotactic cues driven by the tumour microenvironment in HPV-negative OPSCC is different from those in HPV-positive OPSCC and this may directly account for any differences observed in tumour-associated leukocyte sub-populations. Here we examine the abundance of leukocyte populations in HPV-positive and HPV-negative OPSCC and show for the first time that TAN are preferentially recruited to HPV-negative OPSCC. Moreover, using a 3D culture system consisting of HPV-negative or HPV-positive OPSCC spheroids embedded in a tonsillar fibroblast-populated stromal matrix to mimic the tumour microenvironment, we show that both chemokine secretion and neutrophil recruitment are dependent on IL-1β/IL-1R paracrine signalling. Our data point to possible intervention strategies to inhibit TAN recruitment to tumours that may be beneficial for patient prognosis.

## Materials and Methods

### Human Samples

This study utilised patient samples and associated clinical data within the period of primary diagnosis 2002–2012. Patients were selected using the Chemocare database system (National Health Service, UK) and further cross-referenced against the histopathological database held at Sheffield Teaching Hospitals NHS Trust, UK in order to confirm diagnosis and to identify each tissue biopsy reference number. Patients were included in the study if there was sufficient tissue remaining after clinical histopathological diagnosis for TMA generation and if there was a reasonably complete dataset of associated clinicopathological information. All biopsy material was obtained before commencement of anti-cancer treatment. Paraffin-wax embedded tissue samples were retrieved and collated in a blinded fashion with respect to HPV status. HPV positivity using RNAScope® was determined as described in Hendawi et al. [14], whilst expression of p16 staining was assessed using the H-score system with a 300-point cut-off for positivity [14, 15]. Overall HPV status in the samples was found to be proportionate to the UK prevalence of HPV OPSCC, reflecting that a representative population sample was used in this study. Clinical data regarding tumour recurrence, clinical status and follow-up were retrieved from the patient's medical files at the Sheffield Teaching Hospital NHS Foundation Trust, UK. Overall survival was determined by the difference between the date of treatment and either the date of death due to the tumour or last follow-up. The study was conducted with National Research Ethical Committee approval (UK 12/LO/2018).

### Tissue Microarray Construction, Immunohistochemistry, and Image Analysis

Tissue microarrays (TMA) were constructed by selecting tumour regions displaying more than 70% cellularity with minimal necrosis and marked on haematoxylin and eosin-stained sections. TMA were constructed using a tissue arrayer with 3 ×1.0 mm diameter cores from each tissue block arranged at mapped locations into recipient paraffin blocks. TMA were sectioned (5 μm) by microtome onto Superfrost™ adhesive glass slides (ThermoFisher Scientific) and automated immunohistochemical staining performed for CD3 (T lymphocytes, Agilent, clone F7.2.38), CD68 (macrophages, Agilent, clone KP-1) and myeloperoxidase (MPO, Agilent, code IS511,) by the Histology Department, Sheffield Teaching Hospitals NHS Foundation Trust. Stained TMA sections were imaged using an Aperio ScanScope CS slide scanner (Aperio Technologies, Vista, USA). Digital analysis was performed using QuPath software (https://qupath.github.io) [16]. Further details of the QuPath analytical approach is provided in Supplementary Figure 1. Cores with insufficient histology (<80% area) were not included in QuPath analysis. Each OPSCC case was represented by at least two cores in QuPath analysis and the image analysis data was calculated as the mean percent number of positively stained cells as a proportion of the total number of cells in each core. Leukocytes within blood vessels were excluded from the analysis.

### Cell Culture

The OPSCC HPV-positive cell line UPCI-SCC90 was provided by Prof. Susanne Gollin, University of Pittsburgh and the HPV-negative cell line FaDu was purchased from the American Type Culture Collection. The authenticity of cell lines was verified by short tandem repeat analysis and their HPV status confirmed by the HPV Cytology Screening Unit using the cobas® qPCR HPV testing kit that detects a 200 bp region within the L1 region of the HPV genome (Sheffield Teaching Hospital NHS Foundation Trust, UK). Normal tonsillar fibroblasts (NTF) were isolated from biopsies obtained from tonsillectomies at the Sheffield Teaching Hospitals NHS Foundation Trust with written, informed consent (ethical approval 09/H1308/66) as described previously [17]. For this study, NTF isolated from one donor was used in all experiments to limit experimental variation. UPCI-SCC90 cells and NTF were cultured in Dulbecco's Modified Eagle's Medium and FaDu in RPMI-1640, both media were supplemented with 10% v/v foetal calf serum (FCS), 2 mM L-glutamine, 100 IU penicillin and 100 μg/ml streptomycin, (all medium and supplements from Sigma-Aldrich) and cells cultured in a humidified incubator with 5% CO_2_ at 37°C. All cells were confirmed mycoplasma-free before use in experiments.

### Leukocyte Isolation

Human peripheral blood leukocytes were isolated from the venous blood of healthy donors with written, and informed consent (University of Sheffield ethical approval, 012597) as previously described [18]. The donors were two male and one female, none had received the HPV vaccine, none were taking prescription medication or had been ill in the previous 2 weeks, and all were non-smokers. Blood was anti-coagulated using sterile 3.8% sodium citrate (Sigma-Aldrich, W302600) and centrifuged at 400 g for 20 min to separate the plasma from leukocytes. Mononuclear cells were isolated by Ficoll-Paque Plus (GE Healthcare, GE17-1440-02) density-gradient centrifugation, washed twice with Hank's balanced salt solution (HBSS, ThermoFisher Scientific, 14170112) and erythrocytes removed from neutrophils by hypotonic lysis. Total neutrophils and mononuclear cells were re-combined by re-suspension in RPMI-1640 supplemented with 10% v/v FCS and 2 mM L-glutamine then labelled with CellTracker™ Deep Red dye (ThermoFisher Scientific, C34565) according to the manufacturer's instructions. Cell viability was assessed by trypan blue (Sigma-Aldrich, T8154) exclusion and was >95% by light microscopy.

### 3D Tumour—Stromal *in vitro* Culture Models

Multicellular tumour spheroids (MCTS) were generated as previously described [19]. Briefly, 100 μL of a 1 ×10^5^ cells/mL suspension of either FaDu or UPCI-SCC90 cells were added to each well of a 96-well plate previously coated with 100 μL sterile 1.5% w/v type IV agarose (Sigma-Aldrich, 121852) in serum-free medium. Culture plates were incubated for 2 d in a humidified incubator with 5% CO_2_ at 37°C to allow MCTS formation. To prepare 3D tumour-stromal *in vitro* models, type 1 rat-tail collagen (produced in-house) was mixed with 10 × RPMI-1640, 10% FCS, 2 mM L-glutamine and reconstitution buffer (2.2% NaHCO_3_, 4.8% HEPES, 0.25% NaOH in dH_2_O, all from Sigma-Aldrich) and pH adjusted to 7.4. One millilitre collagen was added to 2 ×10^5^NTF and 60 FaDu or UPCI-SCC90 MCTS, the contents mixed to evenly disperse the NTF/spheroids and the hydrogel allowed to solidify for 1 h at 37°C, 5% CO_2_ in a 24-well plate. A ratio of ~1:3 NTF to tumour cells was used in accordance with our previous 2D work [13]. Once set, 1 mL of serum-containing medium was added and models incubated for a further 24 h. Models were then washed twice with serum-free medium and incubated in the absence or presence of 1 or 10 μg/ml anakinra (Trade name Kineret, Amgen) in 500 μl serum-free medium and further incubated for 24 h. Levels of anakinra were maintained throughout the experiment. Culture medium was then removed and stored at −20°C for further analysis by ELISA.

To generate immune cell-containing 3D tumour-stromal models, 200 μL of CellTracker™-labelled total leukocytes were added to the surface of either FaDu or UPCI-SCC90 3D co-culture models in the absence or presence of 10 μg/ml anakinra, and incubated for 24 h. Models were washed twice with HBSS to remove non-infiltrating leukocytes then total cells dispersed from the models using type I collagenase (2 mg/mL in HBSS, Sigma-Aldrich, SCR103), washed in HBSS, sieved to remove cell aggregates and then fixed with 2% paraformaldehyde. Flow cytometry (FACSCalibur™ with associated CellQuest™ software, BD Biosciences) was used to plot side scatter against fluorescence (650 nm emission) to determine the total number of infiltrating leukocytes into tumour-stromal models. These cells were further gated and side scatter plotted against forward scatter to determine the relative numbers of neutrophils, monocytes and lymphocytes according to their cell size and granularity (see **Figure 5** for gating strategy). Each experiment was performed using the leukocytes isolated from one donor with each test performed in technical triplicates and the entire experiment was repeated three times (*n* = 3) using the blood from a different donor (two male, one female, all non-HPV-vaccinated and non-smokers) on each occasion.

### Cell Viability

Cell viability as measured by metabolism of PrestoBlue™ (ThermoFisher Scientific, P50200) was performed according to the manufacturer's instructions. 3D models were incubated with serum-containing medium for 48 h at 37°C, 5% CO_2_. Models were washed with HBSS before addition of fresh medium containing PrestoBlue™ (1:10 v/v) and then incubated for a further 2 h. Medium was removed and the colour change measured by fluorescence excitation at 560 nm and emission at 590 nm.

### Chemokine Level Quantification

Conditioned medium collected from 3D models in the absence or presence of anakinra was subjected to ELISA for CXCL8, CCL2 (BD Biosciences 555244 and 559017, respectively) and CCL5 (R&D systems, DRN00B) according to the manufacturer's instructions.

### Statistics

All data are expressed as mean ± SD of at least three independent experiments performed in triplicate unless otherwise stated. Statistical analysis was undertaken using GraphPad Prism (v8.4.3, GraphPad Software, San Diego, CA). For demographic and clinical data two-sided Chi-squared or Fisher's exact-test was used for comparison of categorical variables and two-tailed Student *t*-test was used for continuous variables. For TMA data pairwise comparisons were performed using Mann-Whitney *U*-test, and for ELISA data group-wise comparisons were made using one-way ANOVA with Tukey's multiple comparisons test. Flow cytometric data was analysed using one sample *t* and Wilcoxon-test. SPSS (v22, IBM Chicago, IL) was used to test for HPV-status effects in cumulative survival curves according to the Kaplan-Meier method with comparisons between survival curves made using the log rank test. Differences between groups was considered significant when *p* < 0.05.

## Results

### HPV-Positive Status Is Associated With Improved 5-Year Survival in OPSCC

**Figure 1:**
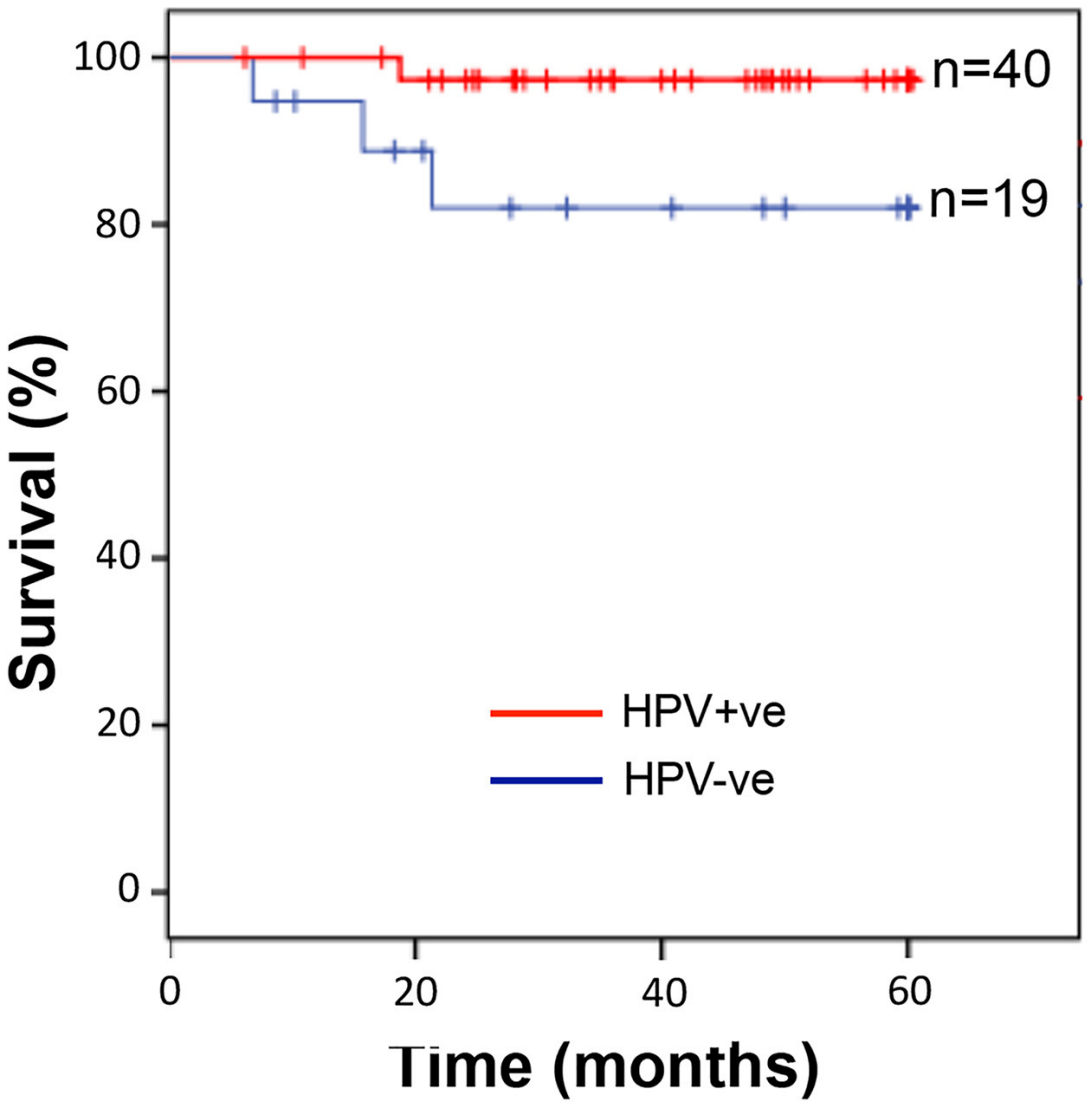
Individuals with HPV-positive OPSCC display significantly better overall 5-year survival than those with HPV-negative OPSCC. Kaplan-Meier analysis followed by log rank-test showed that individuals with HPV-positive OPSCC (*n* = 40) correlated with significantly (*p* = 0.04) improved overall 5-year survival than individuals with HPV-negative OPSCC (*n* = 19).

The patient demographic information is summarised in Supplementary Table 1. The median age was 57 years for HPV-positive and 56 years for HPV-negative patients. There was a statistically significant difference in the levels of alcohol (*p* = 0.018) and smoking (*p* = 0.032) between subjects with HPV-positive and HPV-negative OPSCC. However, there was no significant difference between any of the clinical parameters analysed (Table 1). OPSCC cases were stratified for HPV-status and overall survival over a 5-year follow-up period examined. Kaplan-Meier survival analysis showed that individuals with HPV-positive OPSCC correlated significantly (*p* = 0.04) with better overall 5-year survival than individuals with HPV-negative OPSCC (Figure 1).

**Table 1:** Oropharyngeal squamous cell carcinoma patient demographics and clinical characteristics.

**Characteristics (%)**			**HPV-positive (%)**	**HPV-negative (%)**	***P***
Total Subjects	59 (100)		40 (68)	19 (32)	
Sex	Female	14 (24)	10 (25)	4 (21)	0.739
	Male	45 (76)	30 (75)	15 (79)	
Age (year)	Median		57	56	0.651
	Range		29-68	31-70	
Alcohol^a^	Never		3 (7.5)	0 (0)	**0.018**
	Moderate		22 (55)	6 (31.5)	
	Heavy Unknown		4 (10) 11 (27.5)	7 (37) 6 (31.5)	
Smoking^b^	Smoker		13 (32.5)	9 (47)	**0.032**
	Non-smoker		11 (27.5)	0 (0)	
	Ex-smoker Unknown		9 (22.5) 7 (17.5)	7 (37) 3 (16)	
Site	Tonsil		28 (70)	9 (47)	0.205
	Base of tongue		11 (27.5)	7 (37)	
	Post wall of pharynx		0 (0)	1 (5)	
	Soft palate		0 (0)	1 (5)	
	Oropharynx (not otherwise specified)		1 (2.5)	1 (5)	
Disease stage	Stage I		8 (20)	2 (11)	0.271
	Stage II		16 (40)	5 (26)	
	Stage III		5 (12.5)	6 (32)	
	Stage IV Unknown		10 (25) 1 (2.5)	5 (26) 1 (5)	
T-Stage^c^	T1/T2		24 (60)	7 (37)	0.155
	T3/T4 Unknown		15 (37.5) 1 (2.5)	11 (58) 1 (5)	
N-stage^c^	N0		3 (7.5)	3 (15.7)	0.654
	N1-N2a		28 (70)	13 (68)	
	N2b-N3 Unknown		7 (17.5) 2 (5)	3 (15.7) 0 (0)	
M-Stage^c^			0 (0)	0 (0)	
Grade^c^	Poor		25 (62.5)	9 (48)	0.339
	Moderately		9 (22.5)	8 (42)	
	Well Unknown		2 (5) 4 (10)	1 (5) 1 (5)	
Recurrence	Local		1 (5)	4 (21)	0.495
	Regional None		0 (0) 39 (95)	2 (10.5) 13 (68.5)	

a *Moderate alcohol consumption is defined as up to 10–25 units/week, heavy consumption is defined as >25 units/week.*

b *Ex-smoker is defined as an individual who has smoked at least 100 cigarettes in their lifetime but who had quit smoking within the last 12 months.*

c *Brierly et al. [20].*

*Two-sided Chi-squared or Fisher's exact-test was used for comparison of categorical variables and two-tailed Student t-test was used for continuous variables. P <0.05 are given in bold text*.

### HPV-Negative OPSCC Contain Elevated Numbers of Tumour-Associated Neutrophils Than HPV-Positive OPSCC but Levels of Other Tumour-Associated Leukocytes Are Similar

**Figure 2:**
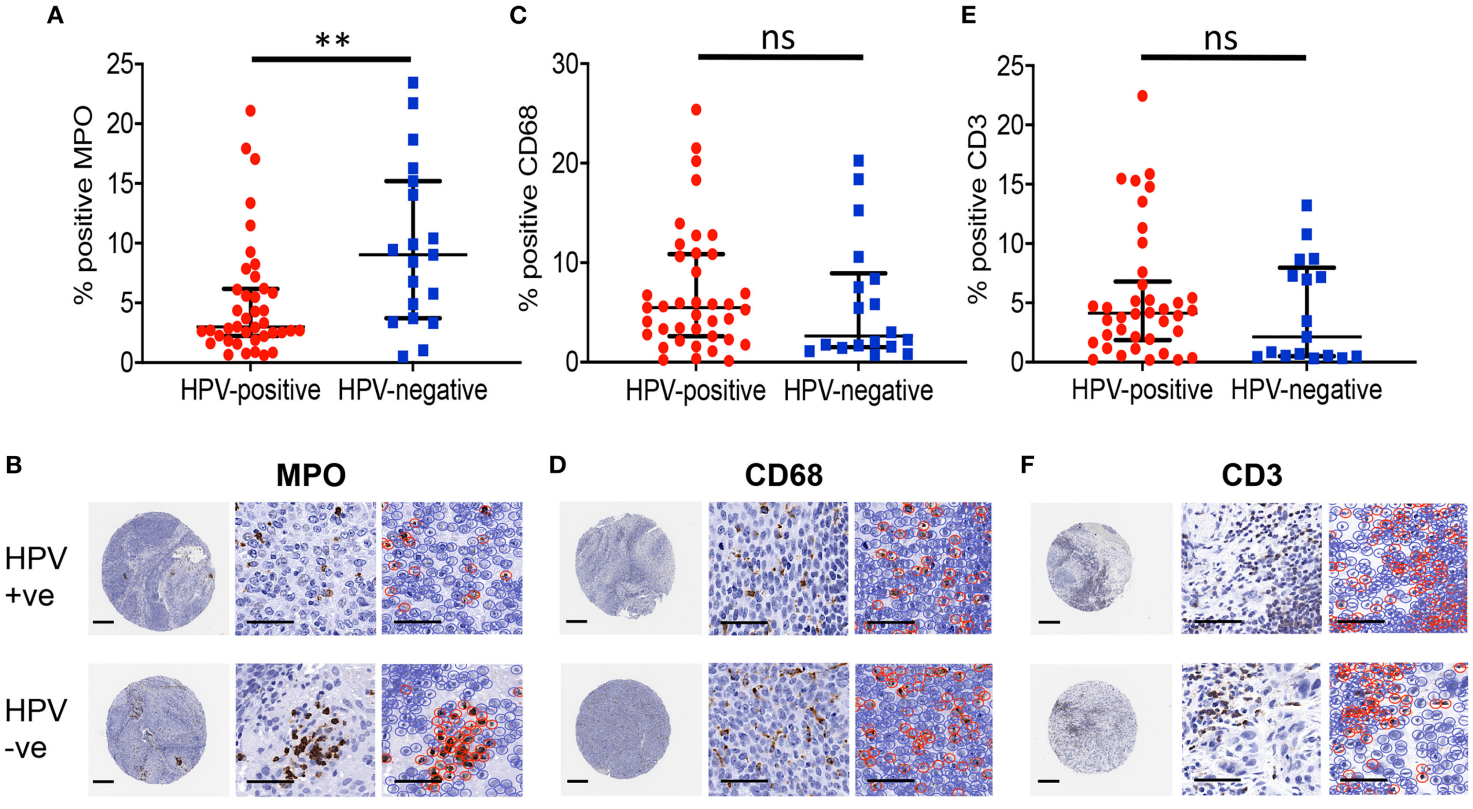
HPV-negative OPSCC contain more neutrophils than HPV-positive OPSCC. Levels of tumour associated-leukocyte populations for HPV-positive and HPV-negative OPSCC tissue sections were analysed by QuPath. **(A,B)** MPO+ neutrophils, **(C,D)** CD68+ macrophages, **(E,F)** CD3+ T lymphocytes. Scale bar = 250 μm for whole cores (left), and 50 μm for magnified core images (centre) and corresponding QuPath image analysis (right). Brown staining shows immuno-positively identified cells within the cores. Data are presented as the median and interquartile range of percent positively stained cells as a proportion of the total number of cells. Differences between groups was determined using a non-parametric Mann-Whitney *U*-test; ^**^p < 0.01.

The number of tumour-infiltrating leukocytes present in HPV-negative or HPV-positive OPSCC was measured by immunohistochemical staining of TMA for myeloperoxidase (MPO; neutrophils), CD68 (macrophages) or CD3 (pan T cells) followed by QuPath image analysis and the percent number of positively stained cells as a proportion of the total number of cells in each core calculated (Supplementary Figure 1). There was a significant (*p* = 0.003) increase in the level of MPO-immunopositive staining in HPV-negative (median 9.04%, *n* = 19) compared with HPV-positive (median 2.98%, *n* = 40) OPSCC, indicating the increased prevalence of neutrophils in HPV-negative compared to HPV-positive OPSCC (Figures 2A,B). In contrast, similar levels of macrophages, identified by CD68-positive staining, were observed in both HPV-positive (median 5.49%, *n* = 39) and HPV-negative (median 2.64%; *p* = 0.277) OPSCC (Figures 2C,D). Likewise, the levels of T lymphocytes were similar in both HPV-positive (median 4.13%, *n* = 38) and HPV-negative (median 2.14%, *n* = 17; *p* = 0.333) OPSCC (Figures 2E,F). The tissue neutrophil to lymphocyte ratio (NLR) for HPV-negative OPSCC was significantly greater (median 2.60) than the ratio for HPV-positive tumours (median 0.773; *p* < 0.01). There were no significant differences observed in the levels of infiltrating leukocyte populations between HPV-positive and HPV-negative OPSCC at disease stage (Supplementary Figure 2) or T-stage (Supplementary Figure 3) examined. Taken together, these data show that HPV-negative OPSCC contained significantly more TAN compared to HPV-positive OPSCC whereas levels of CD68 and CD3-positive leukocyte populations were similar.

### HPV-Negative 3D Tumour-Stromal Culture Models Produce Significantly Higher Levels of Chemokines than their HPV-Positive Counterparts

Our previously published data using a 2D monolayer experimental culture system showed that when cultured with conditioned medium derived from several different HPV-negative OPSCC tumour cells, NTF are stimulated to secrete high levels of chemokines in an IL-1-dependent manner [13]. Here we used a 3D *in vitro* construct containing HPV-negative (FaDu) or HPV-positive (UPCI-SCC90) multi-cellular tumour spheroids (MCTS) (Supplementary Figures 4A–F) embedded in a NTF-populated stromal collagen matrix in an attempt to more accurately model the intimate cell-cell OPSCC tumour-stromal cell interactions within a 3D environment, similar to those occurring *in vivo*. Haematoxylin and eosin stained sections of the tumour-stromal 3D model demonstrated an evenly distributed fibroblast-populated matrix containing MCTS that often display a central necrotic core surrounded by several layers of tumour epithelium (Supplementary Figures 4E,F). The histological appearance of the tumour-stromal 3D model is comparable to that frequently observed with OPSCC tumours *in vivo* (Supplementary Figure 4G) although the density of the fibroblasts within the *in vitro* models appears to be lower than observed *in vivo*.

Cell viability within collagen gels was measured for NTF, FaDu or UPCI-SCC90 spheroids alone or in combination. The mean fluorescence readings for all tests containing cells were increased compared to collagen alone showing that cells remained metabolically active and therefore viable whilst embedded in collagen. When in co-culture with NTF, both UPCI-SCC90 and FaDu displayed an additive and significant (*p* < 0.05) increase in fluorescence compared to when the spheroids or NTF were cultured alone, reflecting the synergistic nature of the co-culture system (Supplementary Figure 4H).

**Figure 3:**
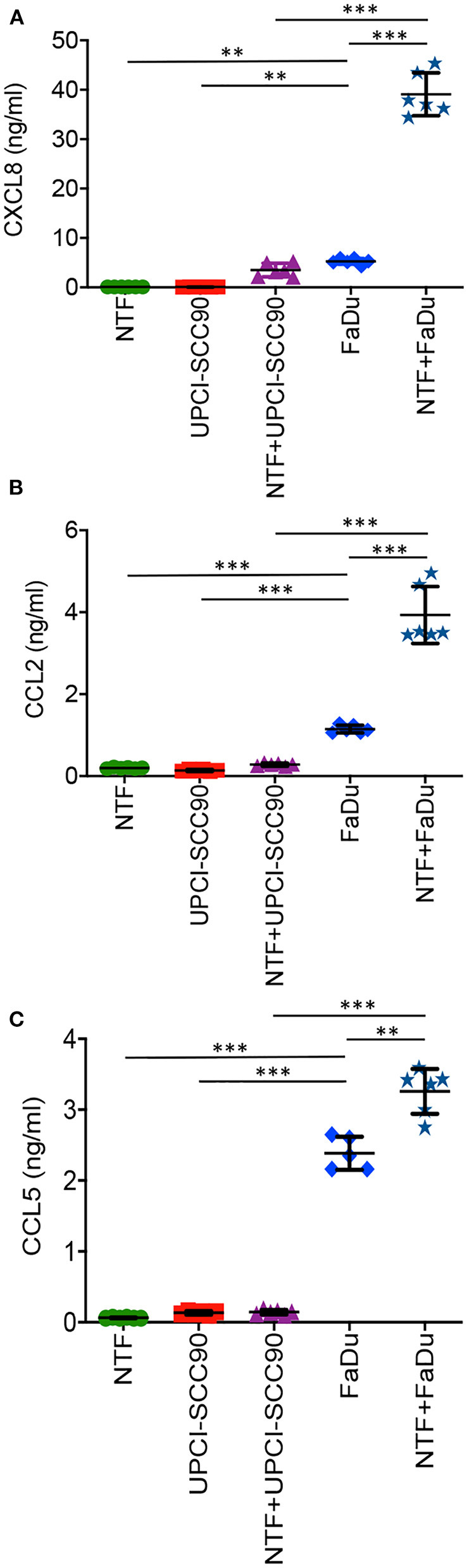
FaDu HPV-negative OPSCC 3D tumour-stromal models display increased chemokine secretion compared to their UPCI-SCC90 HPV-positive counterparts. Chemokine secretion of NTF-populated collagen alone, UPCI-SCC90 and FaDu MCTS alone or NTF/MCTS co-cultures for **(A)** CXCL8, **(B)** CCL2, and **(C)** CCL5. Data are mean ± SD for at least 5 independent experiments. Statistics were performed using One-way ANOVA with Tukey's multiple comparisons test. ^**^p < 0.01, ^***^p < 0.001.

We next measured the levels of chemokines released in models containing NTF or MCTS alone or when in combination. We chose to measure CXCL8, CCL2 and CCL5, as these chemokines are well-recognised, potent chemoattractants for neutrophils, monocytes and lymphocyte populations, respectively. When cultured alone, NTF produced low levels for all three of the chemokines tested with 0.12 ± 0.007 ng/ml, 0.20 ± 0.02 ng/ml and 0.06 ± 0.005 ng/ml for CXCL8, CCL2 and CCL5, respectively (Figures 3A–C). This was also the case for UPCI-SCC90 MCTS when cultured alone (CXCL8−0.09 ± 0.01 ng/ml, CCL2−0.14 ± 0.02 ng/nl and CCL5−0.13 ± 0.001 ± ng/ml; Figures 3A–C). Chemokine secretion was not significantly increased when NTF were co-cultured with UPCI-SCC90 MCTS (CXCL8−3.52 ± 1.38 ng/ml, CCL2−0.28 ± 0.04 ng/nl and CCL5−0.14 ± 0.004 ± ng/ml; Figures 3A–C). In contrast, for CXCL8 (5.28 ± 0.6 ng/ml; *p* < 0.05) and CCL2 (1.15 ± 0.09 ng/ml; *p* < 0.001), FaDu MCTS alone secreted significantly more chemokine than NTF or UPCI-SCC90 MCTS alone, and for CCL5 (2.38 ± 0.23 ng/ml; *p* < 0.001) significantly more than NTF/UPCI-SCC90 co-culture models (Figures 3A–C). However, when FaDu MCTS were combined with NTF in a 3D tumour-stromal model, levels of CXCL8 increased 7-fold (39.1 ± 4.34 ng/ml; *p* < 0.001) and CCL2 by 3-fold (3.93 ± 0.69 ng/ml; *p* < 0.001, Figures 3A,B), showing that NTF and FaDu act synergistically in stimulating production of these chemokines. Levels of CCL5 also increased in the 3D tumour-stromal model compared to MCTS alone, although the difference between the amounts of chemokine released was much less [2.4 ± 0.2 ng/ml compared to 3.3 ± 0.3 ng/ml for FaDu MCTS alone and NTF + FaDu MCTS, respectively (*p* < 0.01); Figure 3C]. These data indicate that OPSCC cells within a 3D environment interact with NTF via paracrine signalling.

### Synergistic Production of Chemokines by HPV-Negative 3D Tumour-Stromal Culture Models Is Mediated by IL-1/IL-1R

**Figure 4:**
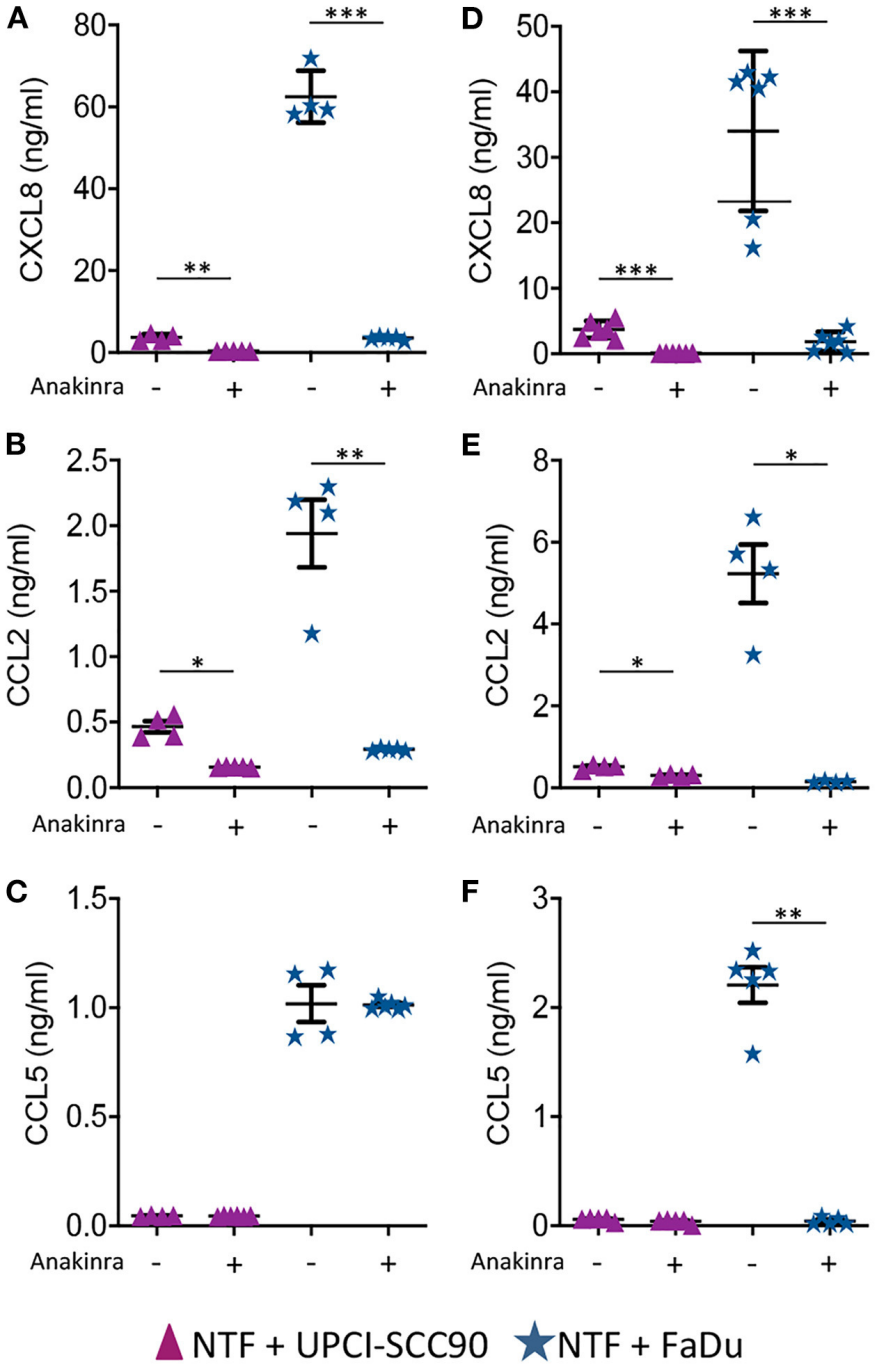
Chemokine secretion by HPV-negative OPSCC 3D tumour-stromal models is reduced by anakinra. **(A)** CXCL8, **(B)** CCL2, and **(C)** CCL5 secretion from HPV-positive UPCI-SCC90/NTF or HPV-negative FaDu/NTF co-cultures in the absence or presence of 1 μg/mL **(A–C)** or 10 μg/mL **(D–F)** anakinra. Data are mean ± SD for at least 4 independent experiments. Statistics were performed using Mann Whitney *U*-test. ^*^p < 0.05, ^**^p < 0.01, ^***^p < 0.001.

We next tested if the IL-1/IL-1R axis was the main paracrine signalling pathway in mediating elevated chemokine release by inhibiting this pathway using anakinra, a highly specific IL-1R antagonist. Here, 3D models containing either UPCI-SCC90 or FaDu MCTS co-cultured with NTF were pre-incubated with 1 or 10 μg/ml anakinra and levels of CXCL8, CCL2 and CCL5 measured in the conditioned medium after 24 h. Pre-incubation with 1 μg/ml anakinra dramatically reduced the production of CXCL8 and CCL2 but not CCL5 for both UPCI-SCC90 and FaDu 3D tumour-stromal models. Specifically, in FaDu-stromal 3D experimental models, CXCL8 was reduced 18-fold (*p* < 0.001) and CCL2 6-fold (*p* < 0.01) upon treatment with anakinra, whereas CCL5 levels remained the same as untreated models (Figures 4A–C). Inhibition of chemokine secretion was even more pronounced when models were pre-incubated with 10 μg/ml anakinra, where levels of CXCL8, CCL2 and CCL5 were almost abolished in anakinra-treated FaDu stromal models (*p* < 0.01; Figures 4D–F).

### HPV-Positive UPCI-SCC90 Tumour-Stromal Models Recruit Fewer Neutrophils than their HPV-Negative FaDu Counterparts Reproducing What Is Observed *in vivo*

**Figure 5:**
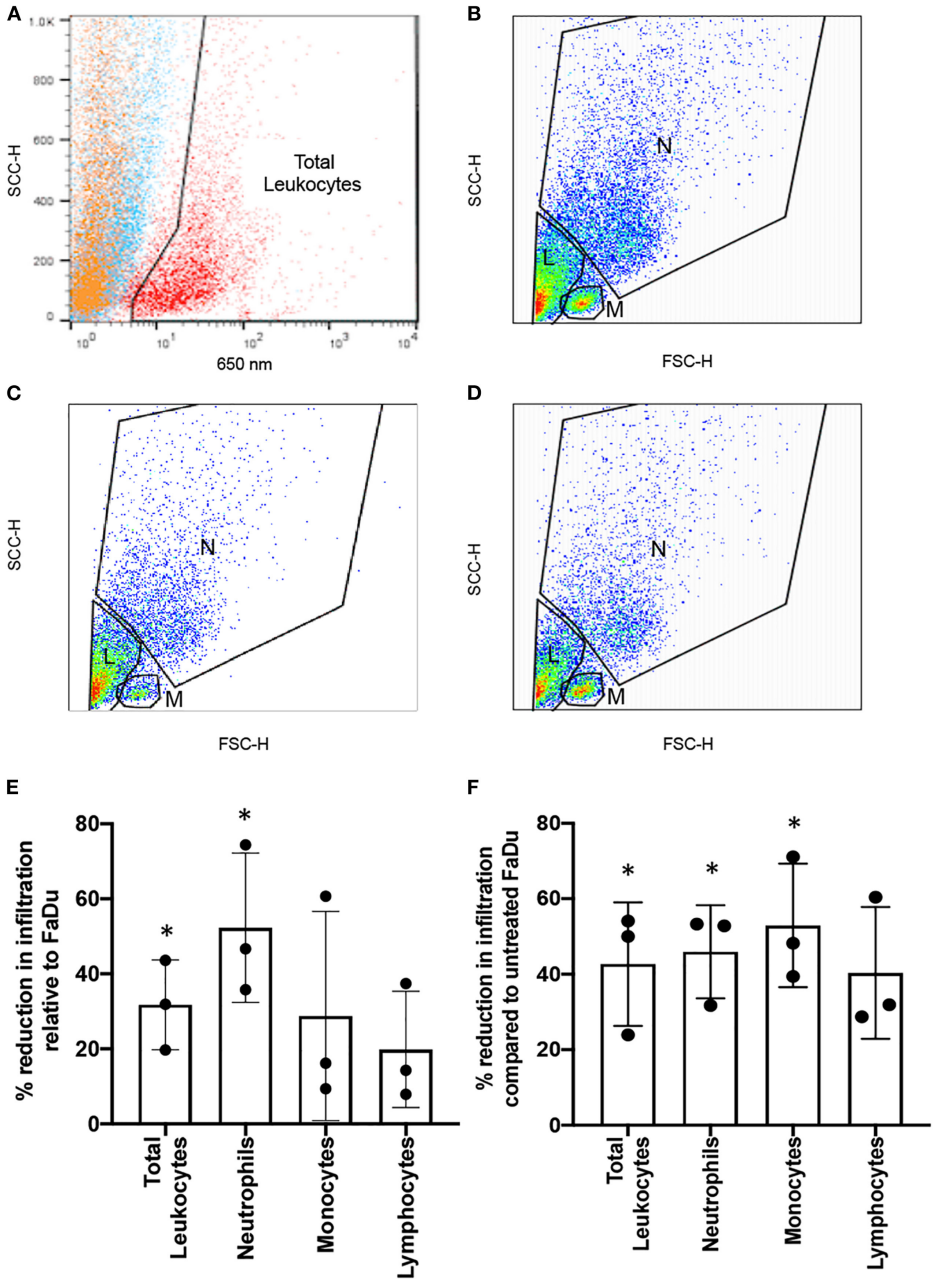
Anakinra reduces leukocyte recruitment in an *in vitro* 3D model of OPSCC. Infiltration of leukocytes into 3D OPSCC tumour-stromal models in the absence or presence of 10 μg/ml anakinra. **(A)** Fluorescence-based gating strategy to identify leukocyte population (red) compared to tumour (orange) or NTF (blue) cell populations. Total leukocytes identified in panel A were re-gated for their well-characterised side and forward scatter properties to determine the number of neutrophils (N), monocytes (M) and lymphocyte (L) populations infiltrating into **(B)** FaDu tumour-stromal models and **(C)** UPCI-SCC90 tumour-stromal models in the absence of anakinra, or **(D)** FaDu tumour-stromal models pre-treated with 10 μg/ml anakinra for 24 h before addition of leukocytes. **(E)** Percent reduction in the number of leukocytes infiltrating HPV-positive UPCI-SCC90 tumour-stromal models relative to those infiltrating HPV-positive FaDu tumour-stromal models. **(F)** Percent reduction in the number of leukocytes infiltrating anakinra treated (10 μg/ml) HPV-negative FaDu tumour-stromal models compared to untreated FaDu tumour-stromal model controls. Data in E and F are mean ± SD for 3 independent experiments with each experiment being performed using the blood from an individual donor. Statistics were performed using one sample *t* and Wilcoxon-test. ^*^p < 0.05.

Fluorescently labelled leukocytes isolated from whole blood were added to HPV-positive UPCI-SCC90 or HPV-negative FaDu tumour stromal models and the levels of total infiltrating leukocytes, as well as individual leukocyte subsets, quantified by flow cytometry using a gating strategy to identify neutrophils, monocytes and lymphocytes based on their well-characterised forward and side scatter profiles (Figures 5A–D). There were 36.8 ± 14.9% fewer total leukocytes infiltrating into HPV-positive UPCI-SCC90 tumour-stromal models compared to those infiltrating into the HPV-negative FaDu tumour-stromal models (*p* = 0.05; Figure 5E, data showing the actual numbers of total leukocytes or leukocyte subsets infiltrating into the tumour-stromal models for each individual experiment is provided in Supplementary Table 1). When broken down into leukocyte subsets, the proportion of neutrophils infiltrating into HPV-positive UPCI-SCC90 tumour-stromal models was 52.3 ± 19.9% less (*p* = 0.045) when compared to those infiltrating HPV-negative FaDu tumour-stromal models, whereas, although overall decreased, the proportion of infiltrating monocytes (28.8 ± 27.9%) and lymphocytes (19.8 ± 15.5%) were not statistically different between UPCI-SCC90 and FaDu models (Figures 5B,C,E). Although these flow cytometric results have a relatively large standard deviation due to donor-to-donor variability, the data reflect those observed in the immunohistochemical analysis of leukocyte infiltration in the patient OPSCC tumour sections, where greater levels of tumour-associated neutrophils were observed in HPV-negative compared to HPV-positive tumours. Since pre-treatment with anakinra significantly reduced the levels of chemokines (CXCL8 and CCL2) in both FaDu and UPCI-SCC90 tumour-stromal models (Figure 4), we reasoned that these anakinra-treated tumour-stromal models would therefore also recruit fewer leukocytes. This was indeed the case with anakinra treatment reducing the numbers of infiltrating leukocytes for both UPCI-SCC90 and more dramatically for FaDu tumour-stromal models (Supplementary Figure 4). Here, anakinra-treated FaDu tumour-stromal models recruited 45.2 ± 12.0% (*p* = 0.023) fewer total leukocytes than untreated models (Figures 5B,D,F). At the leukocyte subpopulation level, the proportion of neutrophils and monocytes infiltrating FaDu tumour-stromal models was significantly reduced by 45.9 ± 12.3% (*p* = 0.023) and 52.9 ± 16.4% (*p* = 0.030) respectively, upon anakinra treatment, whereas the proportion of lymphocytes infiltrating into anakinra-treated FaDu tumour-stromal models was not significantly different (40.3 ± 17.5%; *p* = 0.057) from untreated models (Figure 5F).

## Discussion

The tumour microenvironment plays a major role in directing the course of tumour progression, and factors that regulate the immune response and direct the infiltration of tumour-associated leukocytes are key to this process [21]. It is important to understand the overall tumour-associated immune burden and mechanisms of how this is controlled because several lines of evidence show that the abundance of certain leukocyte subsets within tumours correlate with either poor or improved prognosis.

Although our patient cohort was relatively small, HPV status still stratified the patient groups in terms of outcome, where patients with HPV-positive OPSCC displayed significantly improved 5-year survival than those with HPV-negative tumours, as observed in several other studies [2, 6, 22, 23]. The percent 5-year overall survival for HPV-positive patients seen in this study is similar to those observed in previous studies, whereas we observed an increased 5-year overall survival for HPV-negative OPSCC subjects compared to previous studies [2, 6, 22, 23]. This is likely due to the relatively small sample size for the HPV-negative cohort used in this study compared to other larger studies as well as differences in local detection and/or treatment regimens. Upon leukocyte tumour burden assessment, we found TAN to be significantly more abundant in HPV-negative than HPV-positive tumours; the first time that differences in TAN levels in OPSCC has been shown to correlate with HPV status. We used a validated antiserum specifically raised against neutrophil MPO to detect the presence of TAN, a biomarker commonly used by diagnostic pathology laboratories for the detection of neutrophils. MPO may not be as specific as less commonly used markers such as CD15 and CD66b, although these biomarkers too are expressed by other granulocytes. Unfortunately, we were unable to correlate TAN levels with prognosis for HPV status cases due to the sample size. However, similar data have been reported for OSCC [24], although this form of cancer rarely harbours oncogenic HPV [25]. Here, Li et al., observed increased abundance of CD15+ neutrophils in HPV-negative compared to HPV-positive OSCC, as well as finding that high levels of neutrophils correlated with poor 5-year survival, increased lymph node metastasis and were an independent prognostic factor for OSCC [24]. Using The Cancer Genome Atlas (TCGA) data set Chen et al., also found a higher neutrophil genetic imprint for HPV-negative compared to HPV-positive head and neck cancer [26].

In other tumour types, high levels of TAN have been shown to drive tumour progression by producing factors such as reactive oxygen species that initiate further DNA mutations [27], and by secreting potent pro-tumour factors such as vascular endothelial growth factor and matrix metalloproteinases that promote tumour angiogenesis [28, 29], and transforming growth factor beta (TGF-β) that induces myofibroblast formation [30]. Moreover, neutrophils that display an immunosuppressive phenotype upon stimulation by tumour micro-environmental cues [so called polymorphonuclear myeloid-derived suppressor cells (PMN-MDSC)] have been shown to accumulate in both patient biopsies and murine experimental models of head and neck squamous carcinoma (HNSCC), where they inhibit the tumouricidal functions of natural killer cells by secretion of TGF-β, nitric oxide and arginase-1 [31, 32]. Given these findings, it could be speculated that high TAN levels and their associated secreted factors are key in driving tumour immunosuppression, progression, metastasis and therefore poor outcome in HPV-negative OPSCC. Investigation of the TAN phenotypes in HPV-negative and HPV-positive OPSCC in future studies is warranted.

Similar to TAN, increased levels of TAM have been shown to correlate with poor prognosis in several tumours including HPV-negative OSCC [9, 33]. Although we observed high levels of TAM in tumours there was no significant difference in their abundance between HPV-positive and HPV-negative OPSCC. Our data is in line with other studies reporting similarities in overall TAM levels and HPV status in OPSCC and HNSCC [7, 26, 34]. In contrast, Seminerio et al., found increased prevalence of CD68+ macrophages in HPV-positive compared to HPV-negative OPSCC in the intra-epithelial but not stromal tumour component [33]. It is possible that different TAM phenotypes are recruited to OPSCC. Although we did not examine TAM polarisation status, a recent study found a higher M1/M2 TAM ratio in HPV-positive HNSCC that may account for differences [26].

Much attention has been recently paid to TIL that are correlated with improved prognosis in many tumours [35] and are the basis for several current immunotherapy approaches that have had variable success in treating head and neck cancers [36]. Using CD3, a pan T cell marker, we found no difference in the levels of CD3+ lymphocytes in HPV-positive compared to HPV-negative OPSCC in our patient cohort, a similar finding previously observed by others [34, 37]. However, there is now compelling evidence that it is the recruitment of specific lymphocyte subsets that are crucial in driving anti-tumour responses, in particular CD8+ T cells, where the ability to control HPV-positive tumours is likely mediated by CD8+ T cell-specific recognition of HPV-derived antigens [38, 39]. Indeed, numerous reports have shown increased abundance of CD8+ T cells in HPV-positive compared to HPV-negative OPSCC where their presence is correlated with overall improved survival [5–7, 37, 40, 41]. In addition, CD20+ B cells [34, 40, 42] and FoxP3+Treg [5, 34] are also increased in HPV-positive compared to HPV-negative OPSCC, suggesting a complex interplay in adaptive immunity in response to HPV-driven oncogenesis. In light of this substantial evidence we did not pursue further lymphocyte characterisation at the sub-population level.

The ratio of circulating peripheral blood neutrophils in relation to lymphocytes has been suggested as a potential prognostic marker for many cancers including OPSCC [43, 44]. A high circulating neutrophil to lymphocyte ratio (NLR) has been found for both HPV-positive and HPV-negative OPSCC [45]. In HPV-positive OPSCC, a high NLR was associated with higher T classification, lower 5-year overall survival and disease-free survival [46]. Similarly, in a large cohort study, Huang et al. found higher levels of circulating neutrophils in HPV-negative compared to HPV-positive OPSCC, although both correlated with lower overall survival compared to patients with lower numbers of circulating neutrophils [47]. Contrary to this, high circulating lymphocyte levels were associated with improved recurrence-free survival in HPV-positive OSCC. However, following multivariate analysis the investigators found that a high circulating neutrophil count predicted for lower overall and recurrence-free survival for only the HPV-positive cohort [47]. In contrast, Rosculet et al. found that NLR was an indicator for both recurrence-free and overall survival in OPSCC but this association was lost when HPV-status was included in the analysis [48]. In support of this, a high NLR predicted for a worse 5-year overall survival compared to a low NLR in HPV-negative OPSCC [49]. Moreover, Rachidi et al. found that the circulating NLR was significantly lower in HPV-positive OPSCC compared to the HPV-negative counterparts [50].

This study showed that the tissue NLR is significantly greater for HPV-negative than HPV-positive OPSCC, which is supportive of some but not all of the current circulating NLR data. There may be a discourse between the numbers of circulating leukocytes and those recruited to the tumour micro-environment particularly for lymphocytes, as it appears that HPV-positive OPSCC contain greater numbers of specific subsets (CD8+ T cells, CD20-B cells and FoxP3+Treg) than their HPV-negative counterparts that are also associated with overall improved survival in HPV-positive OPSCC [5–7, 37, 40, 41]. Larger scale tissue-based immunohistochemical analysis in concert with measurement of circulating NLR is required to resolve the suitability of this type of analysis for prognostication of OPSCC with HPV-status.

Given the striking increase in TAN numbers in HPV-negative compared to HPV-positive tumours, we decided to examine the mechanisms by which the disparity in TAN abundance may be mediated. Recruitment of leukocytes to tumours is driven by chemokines; with specific chemokines responsible for the recruitment of particular leukocyte subsets and several studies have reported that OPSCC cells express a number of chemokines [13, 41, 51]. We reasoned that HPV-negative tumours would produce more neutrophil-specific chemokines than HPV-positive tumours as a mechanism to recruit increased numbers of TAN. Indeed, using a rudimentary 2D culture medium transfer model we previously showed that NTF were stimulated to secrete elevated levels of several cytokines in response to the culture medium from a number of HPV-negative but not HPV-positive OPSCC cell lines [13, 52]. Moreover, this increase was mediated in an IL-1-dependent manner with IL-1 liberated from HPV-negative OPSCC cell lines acting on the NTF IL-1R to stimulate chemokine production [13]. However, traditional 2D-based monolayer cell culture systems lack the 3D architecture and the spatial complexity that give solid tumours their characteristic features such as gradients for oxygen, pH, nutrition and waste products that give rise to areas of tumour necrosis and hypoxia, and so data generated in 2D often does not reflect what occurs in 3D. Most tumours grow as 3D masses closely surrounded by a fibroblast-populated stroma that communicate with tumour and other cells via paracrine signalling, driving tumour progression. *In vivo* orthotopic tumours cannot be used because the oropharyngeal cavity of rodents is small and any sizable tumour grown at this site would cause rapid asphyxiation and so *in vivo* models of OPSCC are limited to subcutaneously grown tumours that are far removed in terms of tissue structure and microenvironment than the tumours observed in humans. In addition, HPV is not a natural host of rodents and so an inappropriate immune response may ensue giving rise to false-positive data. We therefore endeavoured to recreate the tissue architecture using tumour spheroids [structures that are known to replicate many biophysical features of avascular tumours, [53]], embedded in a NTF-populated collagen matrix to more closely mimic the *in vivo* OPSCC microenvironment.

Using these 3D tumour-stromal models we observed that HPV-negative FaDu tumour/NTF co-cultures produced substantially more chemokines than NTF or FaDu MCTS cultured alone, and importantly, much more than UPCI-SCC90 HPV-positive tumour-stromal models. Furthermore, expression of the neutrophil-specific chemokine CXCL8 was produced at significantly higher levels than CCL2 or CCL5 in HPV-negative tumour-stromal models, suggesting that neutrophil-specific chemokines may out-weigh those for monocytes and lymphocytes, potentially skewing leukocyte subset recruitment in these tumours. We were unable to produce reliable and consistent MCTS from other HPV-positive cells lines and so our 3D analysis is limited to FaDu and UPCI-SCC90 cell lines. However, the chemokine profiles detected using these MCTS directly correlated with the profiles observed for other HPV-positive/negative cell lines in our previous 2D study [13], providing some evidence that these phenomena is not cell line specific but may be a general occurrence for HPV status. In support of this, analysis of the Gene Expression Omnibus (GEO) and TCGA databases showed significantly elevated levels of CXCL8 in HPV-negative compared to HPV-positive tumours [24], consistent with our *in vitro* 3D model data. In addition to CXCL8, increased abundance of other neutrophil-specific chemokines such as CXCL1, CXCL5 and CXCL6 is likely, as evidenced by our previous cytokine array analysis [13], and this may further skew the imbalance in leukocyte recruitment in favour of preferential neutrophil recruitment.

Blocking IL-1R using the highly specific antagonist anakinra almost completely abolished chemokine production, providing further support to the notion that IL-1/IL-1R paracrine signalling between HPV-negative tumour cells and NTF is of paramount importance for chemokine production. In support of this, increased levels of IL-1β have been found in HPV-negative compared to HPV-positive OPSCC patient samples [13, 41].

Tonsillar [54] and oral keratinocytes [55] constitutively express IL-1 at basal levels, but levels significantly increase upon DNA-induced malignant transformation [56, 57] and this appears to be important in tumour progression, as pharmacological inhibition of IL-1β interrupts chemically-induced oral carcinogenesis in rodent models [57]. HPV infection appears to ablate the ability of keratinocytes to express IL-1β; indeed we previously observed low levels of IL-1α and IL-1β in HPV-positive compared to HPV-negative cell lines and tumour biopsies [13]. Moreover, a complete lack of IL-1β gene expression has also been observed in HPV-positive cervical carcinoma cell lines [56]. In HPV infected cervical keratinocytes, Niebler et al., demonstrated that IL-1β is continually degraded in a HPV-16, E6 driven proteasome-dependent process via ubiquitin ligase, leading to complete loss of the cytokine as HPV-induced malignancy develops [56]. It is plausible that such a mechanism also occurs in HPV-positive OPSCC cells. Since IL-1β is known to drive NF-κB-induced chemokine expression in many cell types, it is logical that HPV-negative OPSCC are driven to secrete high levels of chemokines and therefore recruit increased numbers of TAN, whereas chemokines are much less abundant in HPV-positive OPSCC and so TAN recruitment is reduced. Li et al. overexpressed HPV18 E7 in the HPV-negative adenosquamous OSCC cell line Cal27 and showed that this directly reduced CXCL8 gene expression and protein secretion, suggesting that HPV gene products may have a more direct effect on chemokine production [24], although such a mechanism has not been shown for OPSCC cells. HPV infection may act to suppress TAN recruitment to HPV-positive OPSCC, although more research is required to further define this link.

Given the high levels of chemokines produced by the paracrine tumour-stromal interactions in HPV-negative OPSCC models it was no surprise that these co-cultures exhibited substantially more overall leukocyte recruitment than HPV-positive 3D models. However, it is remarkable that we observed significantly more neutrophil infiltration into HPV-negative tumour-stromal models than their HPV-positive counterparts, mirroring that observed in human patient tumour samples. Treatment with anakinra significantly reduced the recruitment of both neutrophils and monocytes into both HPV-positive and HPV-negative tumour-stromal models, although the treatment was far more pronounced for HPV-negative tumour-stromal models, once again underscoring the importance of IL-1β in immune cell recruitment to tumours. These data are encouraging and validate the usefulness of human 3D *in vitro* models to study complex tumour microenvironment interactions with multiple cell types that may negate some of the problems experienced in the differences between human and murine immune systems encountered during *in vivo* animal experiments and also with 2D culture.

The use of anakinra to inhibit immune cell infiltration is an interesting concept. Indeed, anakinra was found to reduce circulating levels of CXCL8, tumour growth and the number of infiltrating TAM into mice bearing erlotinib-resistant adenosquamous OSCC (Cal27) and laryngeal carcinoma (SQ20B) xenografts grown subcutaneously. Overall survival in these mice was increased further when they were treated with the epidermal growth factor receptor (EGFR) tyrosine kinase inhibitor, erlotinib, indicating that this adjunct therapy may be effective at overcoming EGFR inhibitor tumour resistance [58]. Anakinra has also been shown to reduce the levels of CXCL8 and number of TAM as well as reducing lymph node metastasis in a murine model of lung cancer [59]. Whereas, in breast cancer blocking IL-1 activity with anakinra or the IL-1β specific antibody, canakinumab, reduced breast cancer metastasis by inhibiting epithelial to mesenchymal transition and preventing metastatic outgrowth of disseminated tumour cells via inhibition of wnt signalling pathways [60–62]. Since blockade of IL-1R by anakinra reduced leukocyte levels in both HPV-positive and HPV-negative OPSCC *in vitro*, it could be argued that this antagonist may be effective for both types of tumour, however, the increased numbers of leukocytes recruited to HPV-negative OPSCC suggests that the treatment would be most effective for this cancer type. Therefore, blockade of the IL-1/IL-1R axis may be a promising adjunct anti-tumour therapy that would particularly affect HPV-negative OPSCC, although there may be inherent side effects in the blocking of this key inflammatory pathway, such as potential changes to inflammatory signalling pathways leading to dysregulated host inflammatory responses to infection or alterations to the activation of important immune cells types such as natural killer and cytotoxic T cells that are crucial in host anti-cancer responses [63, 64].

In summary, our human *in vivo* and 3D tumour-stromal *in vitro* data show, for the first time, that HPV-negative OPSCC contain significantly more TAN than HPV-positive OPSCC. Additionally, the mechanism of neutrophil recruitment appears to be via IL-1-mediated CXCL8 release by NTF. HPV infection appears to prevent IL-1 expression by tumour cells thereby suppressing TAN recruitment. Since TAN have been linked with poor prognosis, their apparent reduced recruitment to HPV-positive tumours may partially explain the improved outcome imparted by HPV-infection in OPSCC. Targeting the IL-1/IL-1R axis may be a viable consideration for the treatment of HPV-negative OPSCC.

## Data Availability Statement

The raw data supporting the conclusions of this article will be made available by the authors, without undue reservation.

## Ethics Statement

The studies involving human participants were reviewed and approved by National Research Ethical Committee approval (UK 12/LO/2018). Written informed consent for participation was not required for this study in accordance with the national legislation and the institutional requirements.

## Author Contributions

CM, KH, and RB conceived and designed the research. SA-S, NH, BO, and CM performed experiments, analysed the data, conducted statistical analysis, and interpreted the results. The manuscript was written and figures prepared by CM, SA-S and BO, and further edited by all the authors. PO contributed essential reagents and expert knowledge. All authors are aware of the content and have read and approved the manuscript for publication.

## Conflict of Interest

The authors declare that the research was conducted in the absence of any commercial or financial relationships that could be construed as a potential conflict of interest.
