# Supplementary material for: Increased Abundance of Tumour-Associated Neutrophils in HPV-Negative Compared to HPV-Positive Oropharyngeal Squamous Cell Carcinoma Is Mediated by IL-1R Signalling
Source: Front Oral Health. 2021 Feb 11;2:604565. doi: 10.3389/froh.2021.604565 (PMC8757728; doi:10.3389/froh.2021.604565)
Supplement: Supplementary file 1 [file Data_Sheet_1.PDF]

Supporting Information

Increased abundance of tumour-associated neutrophils in HPV-negative compared to HPV-positive oropharyngeal carcinoma is mediated by IL-1R signalling

Sarmad Al-Sahaf[1], Naeima B. Hendawi[1], Bethany Ollington[1], Robert Bolt[1], Penelope D. Ottewell[2], Keith D. Hunter[1], Craig Murdoch[1]*

[1]School of Clinical Dentistry, 19 Claremont Crescent, University of Sheffield, S10 2TA, UK
[2]Department of Oncology & Metabolism, Medical School, Beech Hill Road, University of Sheffield, S10 2RX, UK

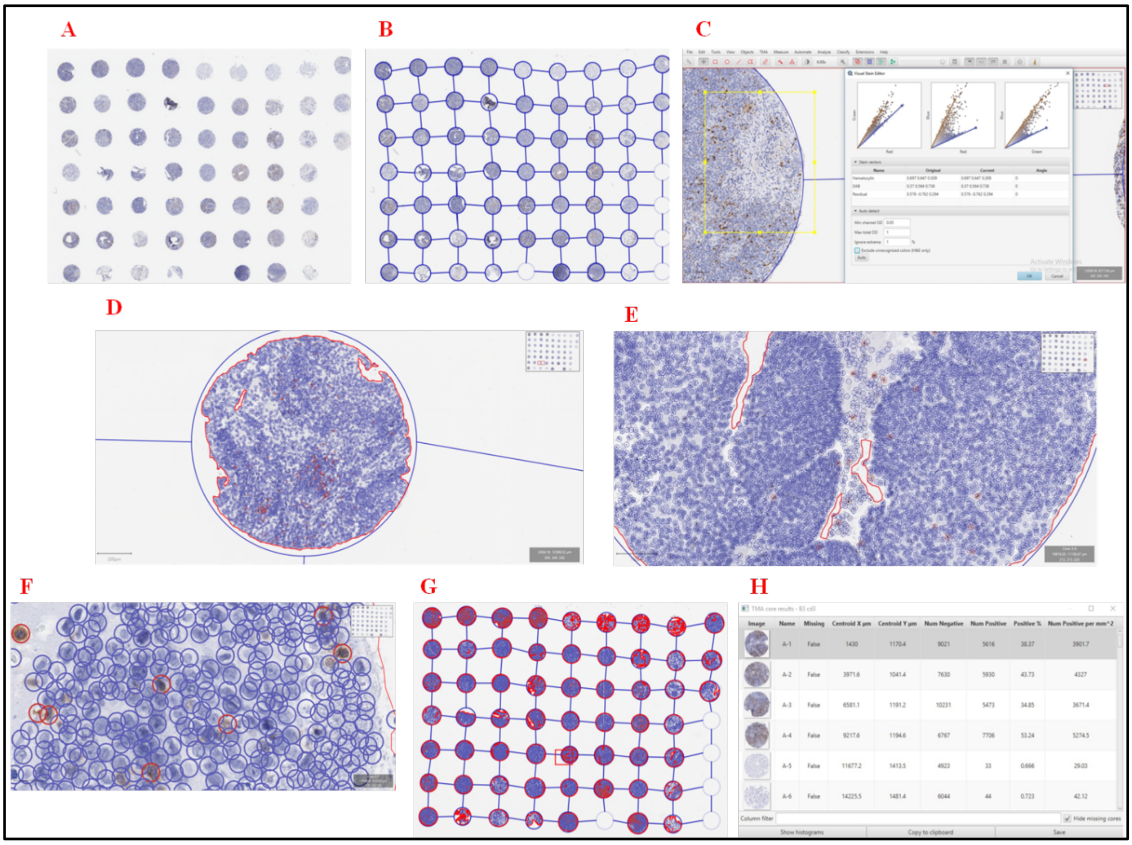

Supplementary Figure 1. QuPath analysis pathway used to interrogate the immunohistochemically stained TMA. A) TMA were scanned using Aperioscan and the digital images imported into the QuPath program and displayed on the computer screen. Each biomarker (e.g. MPO, CD68, CD3) was analysed separately. B) Digitized core images were then de-arrayed by creating an image capture circle of a standard 1.2 mm^2 around the circumference of each 1 mm^2 core. Missing cores or cores with less than 80% cell coverage were not included. C) Immunostain (brown DAB staining for all cell markers) estimation was performed on a representative core in the TMA. A representative area was chosen that included immuno-positively stained cells, background and strong or weak haematoxylin staining, and these criteria were used to train the image analysis using the '*analyse, processing and estimate stain vectors*' function in the QuPath software. D) Tissues were then distinguished via thresholding to mark

tissue boundaries (magnified further in E). The data at this stage was cleaned further via morphological processing using the ‘*analyse, pre-processing, simple tissue detection*’ function in QuPath. The values for the threshold and pixel size were 220 and 4 µm, respectively. F) Individual cells were selected as immuno-positive or immuno-negative by stain separation using ‘*colour deconvolution*’. Peaks representing nuclear haematoxylin-alone stained cells were identified from DAB + haematoxylin stained cells using the ‘*analyse, cell analysis, fast cell count*’ function, along with image smoothing. This allowed the assignment of individual immuno-positive from non-stained cells. G) The number of immuno-positive cells and the number of total haematoxylin nuclear cells in the previously designated core areas (in image D) was then calculated for each individual core on the TMA using the ‘*measure, show TMA, measurements*’ commands in QuPath. H) Data was presented is depicted in image H for all cores and data exported to CSV files for further analysis in Microsoft Excel and GraphPad Prism.

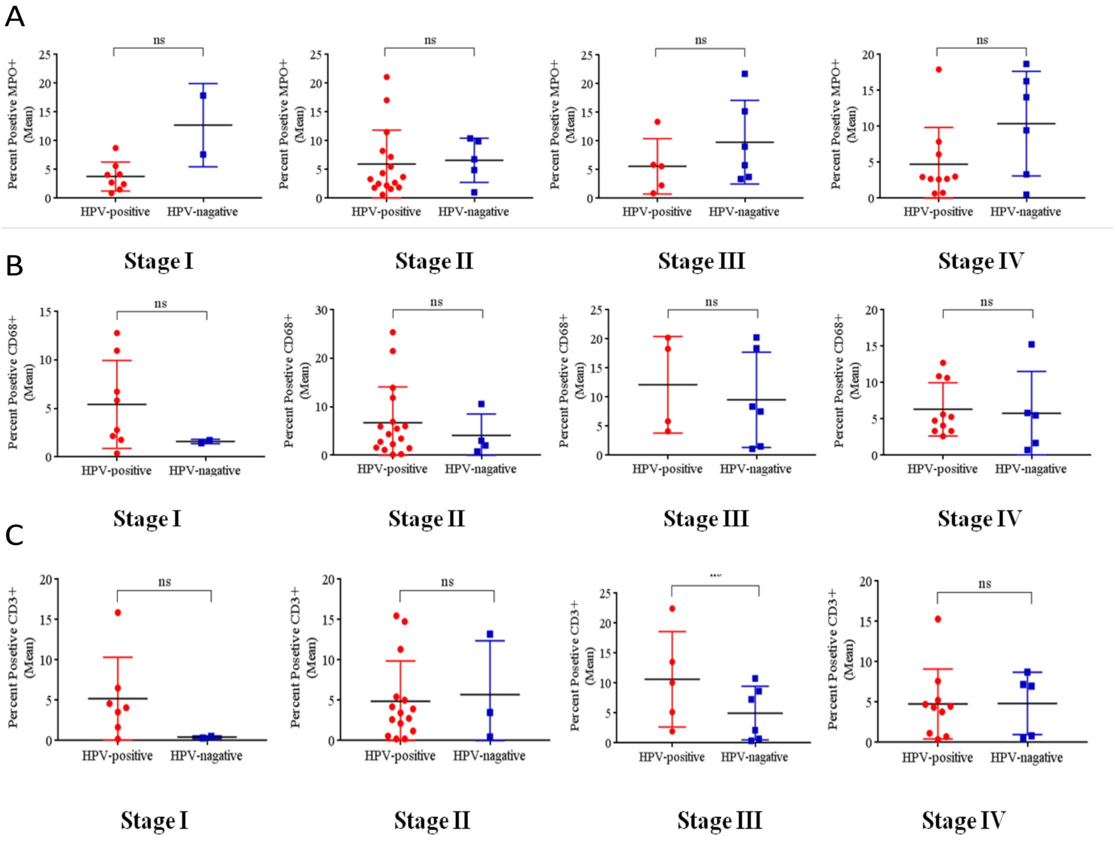

Supplementary Figure 2. Infiltration of tumour-associated leukocytes is not dependent on tumour stage. Number of infiltrating A) MPO+ (TAN), B) CD68+ (TAM) and C) CD3+ lymphocytes in OPSCC according to tumour stage. Data are presented as mean ± SD; statistical analysis was performed using a Mann-Whitney U test.

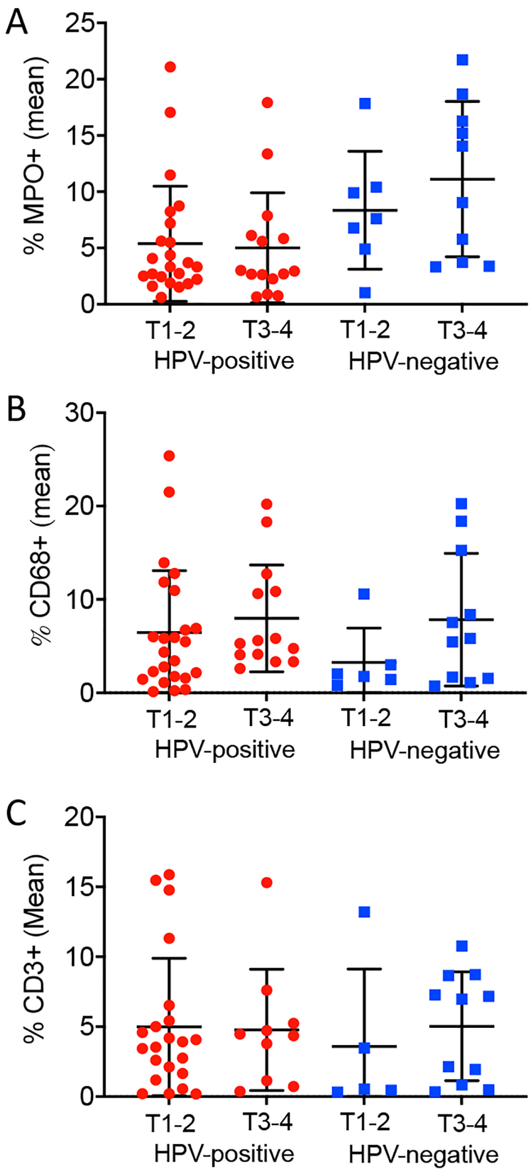

Supplementary Figure 3. Infiltration of tumour-associated leukocytes is not dependent on T-stage. Number of infiltrating A) MPO+ (TAN), B) CD68+ (TAM) and C) CD3+ lymphocytes in OPSCC according to T-stage. Data are presented as mean ± SD; statistical analysis was performed using an ANOVA; no significant differences between datasets were found for any variables tested.

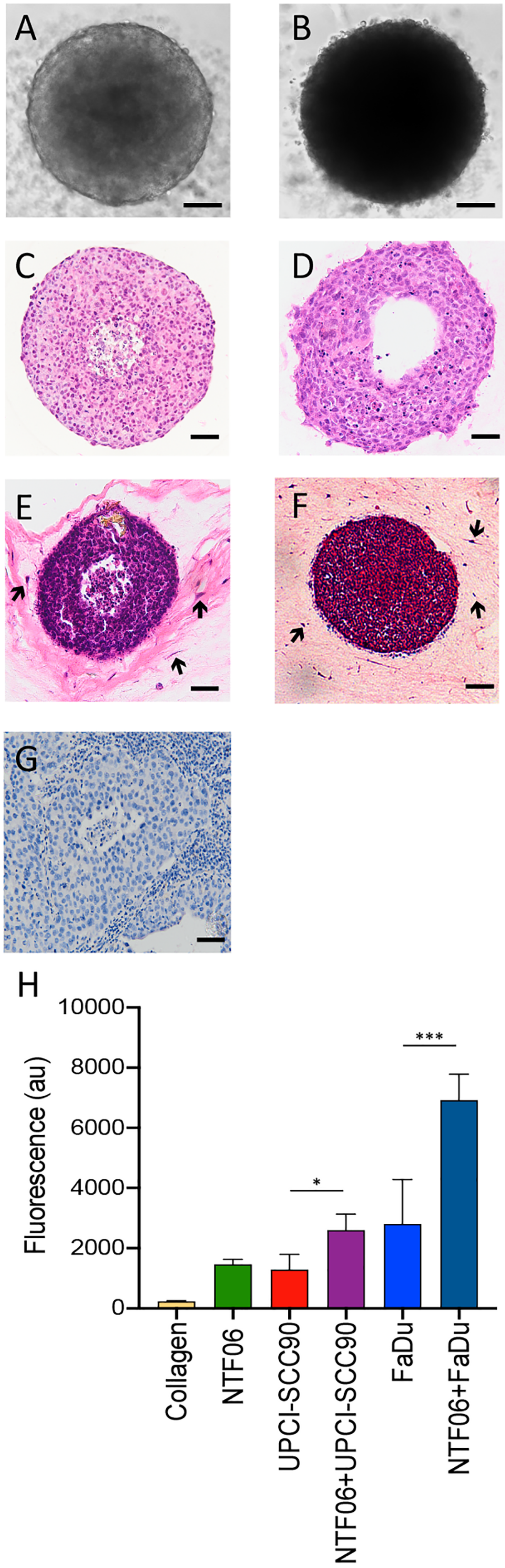

Supplementary Figure 4. Light microscopy images of A) FaDu and B) UPCI-SCC90 MCTS, scale bar = 100 µm. Haematoxylin and eosin stained sections of C) FaDu and D) UPCI-SCC90 MCTS scale bar = 100 µm. E) FaDu and F) UPCI-SCC90 MCTS embedded within a NTF-populated collagen stroma (black arrows point to NTF in panels E and F, scale bar = 100 µm). G) Haematoxylin and eosin stained section of a human HPV-negative OPSCC; scale bar = 50 µm. H) PrestoBlue assay showing the viability of NTF, UPCI-SCC90 and FaDu MCTS, and NTF and MCTS-stromal models within collagen hydrogels. Data are presented as mean ± SD; statistical analysis was performed using an ANOVA; *p<0.05, ***p<0.01.

Supplementary Table 1. Number of total leukocytes and individual leukocyte sub-populations infiltrating into 3D tumour-stromal models after 24 h for each donor used in this study, in the absence or presence of the IL-1R antagonist, anakinra (+AK). FaDu = HPV-negative OPSCC, UPCI-SCC90 = HPV-positive OPSCC, NTF = normal tonsillar fibroblasts. Data are mean values of 3 technical flow cytometry readings.

Donor 1	**FaDu+NTF**	**FaDu+NTF+AK**	**UPCI-SCC90+NTF**	**UPCI-SCC90+AK**
Total Leukocytes	20567	9434	11596	9918
Neutrophils	6633	3132	3533	2938
Monocytes	2746	1663	1079	711
Lymphocytes	9884	3916	6189	5555

Donor 2	**FaDu+NTF**	**FaDu+NTF+AK**	**UPCI-SCC90+NTF**	**UPCI-SCC90+NTF+AK**
Total Leukocytes	3264	1631	2620	1291
Neutrophils	1169	546	750	561
Monocytes	340	176	308	200
Lymphocytes	505	360	433	227

Donor 3	**FaDu+NTF**	**FaDu+NTF+AK**	**UPCI-SCC90+NTF**	**UPCI-SCC90+NTF+AK**
Total Leukocytes	6444	4417	3416	2726
Neutrophils	4934	3372	1265	535
Monocytes	265	76.5	222	179
Lymphocytes	1214	826.5	1118	363
